# Microarray and bioinformatic analysis of conventional ameloblastoma: an observational analysis

**DOI:** 10.1590/1678-7757-2022-0308

**Published:** 2023-01-23

**Authors:** Luis Fernando JACINTO-ALEMÁN, Javier PORTILLA-ROBERTSON, Elba Rosa LEYVA-HUERTA, Josué Orlando RAMÍREZ-JARQUÍN, Francisco Germán VILLANUEVA-SÁNCHEZ

**Affiliations:** 1 Universidad Nacional Autónoma de México Facultad de Odontología División de Estudios de Posgrado e Investigación Ciudad de México México Universidad Nacional Autónoma de México, Facultad de Odontología, División de Estudios de Posgrado e Investigación, Departamento de Patologia y Medicina Bucal, Ciudad de México, México.; 2 Universidad Nacional Autónoma de México Instituto de Fisiología Celular Ciudad de México México Universidad Nacional Autónoma de México, Instituto de Fisiología Celular, Ciudad de México, División de Neurociencias, Ciudad de México, México.; 3 Universidad Nacional Autónoma de México Escuela Nacional de Estudios Superiores Guanajuato México Universidad Nacional Autónoma de México, Escuela Nacional de Estudios Superiores, Guanajuato, México.

**Keywords:** Ameloblastoma, Computational Biology, Platelet-derived growth factor Alpha, IL2RA protein, human

## Abstract

Ameloblastoma is a highly aggressive odontogenic tumor, and its pathogenesis is associated with many participating genes.

**Objective:**

We aimed to identify and validate new critical genes of conventional ameloblastoma using microarray and bioinformatics analysis.

**Methodology:**

Gene expression microarray and bioinformatic analysis were performed using CHIP H10KA and DAVID software for enrichment. Protein-protein interactions (PPI) were visualized using STRING-Cytoscape with MCODE plugin, followed by Kaplan-Meier and GEPIA analyses that were used for the candidate’s postulation. RT-qPCR and IHC assays were performed to validate the bioinformatic approach.

**Results:**

376 upregulated genes were identified. PPI analysis revealed 14 genes that were validated by Kaplan-Meier and GEPIA resulting in PDGFA and IL2RA as candidate genes. The RT-qPCR analysis confirmed their intense expression. Immunohistochemistry analysis showed that PDGFA expression is parenchyma located.

**Conclusion:**

With bioinformatics methods, we can identify upregulated genes in conventional ameloblastoma, and with RT-qPCR and immunoexpression analysis validate that PDGFA could be a more specific and localized therapeutic target.

## Introduction

Odontogenic tumors (OTs) are oral lesions that impact the quality of life of patients because they affect not only the teeth but the maxilla and mandible. OTs constitute a group of heterogeneous diseases ranging from hamartomatous lesions to benign and malignant neoplasms with metastatic potential. These are derived from the epithelium, ectomesenchyme, and/or mesenchymal elements of the odontogenesis apparatus.^1^

The epidemiology of OTs varies throughout the world; in some countries, the most frequent OT is ameloblastoma (Hong Kong, Japan, Zimbabwe, and Nigeria), whereas in others (United States of America, Brazil, and Canada) the most frequent tumor is odontoma.^1,2^ The most common OT in Mexico is odontoma, followed by ameloblastoma, myxoma, adenomatoid odontogenic tumors, and calcifying odontogenic cysts.^3^

Ameloblastoma is a slow-growing locally invasive benign OT with different histological variants, which may be located in the posterior zone of the mandible or maxilla. Because of its potential for recurrence, it is often classified as an aggressive tumor. The estimated global incidence of ameloblastoma is 0.5 cases per million people per year, with the age of diagnosis ranging from 30–60 years. Conventional ameloblastoma (CAm) is the most common variant, followed by unicystic and peripheral ameloblastoma. Although the precise etiology of CAm is unknown, dysregulation of many genes associated with odontogenesis is speculated to play an important role in its histogenesis.^2,4^ Changes in the expression or mutations in genes, such as BRAF, Ras, FGFR2, and SMO, among others, could be associated with its histogenesis.^5,6^ Given this complexity, high-throughput assays offer an alternative to comprehensively analyze this neoplasm. Microarray technology has been used to obtain information on the genetic alterations that occur in several diseases, including neoplasms, such as CAm. Much data are obtained with high-throughput analysis and integrated bioinformatics methods are necessary to unravel the mechanisms underlying the pathogenesis of diseases and to explore and identify novel biomarkers that could help us in further studies.^7,8^ Previous approaches with microarrays assays had shown elements of SHH, cell-cycle regulation, inflammation, MAP kinase pathways, and other molecules, which were confirmed via tests, such as PCR, immunohistochemistry, or NanoString, suggesting that these regulators are important elements of the pathogenesis of conventional ameloblastoma.^9-11^ The bioinformatic analysis is only the first step for new biomarkers to be proposed. Those must be corroborated with particular assays, so that this information can cross over to the clinical level.^12^ The objective was to identify and validate new critical genes of conventional ameloblastoma using microarray and bioinformatics analysis.

## Methodology

### Selection of CAm cases

This study was approved by the Institutional Technical Committee of the Support Program for Research and Technological Innovation Projects (DGAP/1956/2019) of the National Autonomous University of Mexico (UNAM). CAm samples were retrieved from the histopathological paraffin block archives of the Oral Medicine and Pathology Department, Postgraduate Division of Dentistry School (ISO-9001:2015 certified CMX C SGC 157 2017). This study was conducted following the integral privacy notice for patients from the Dentistry School, protecting their identity.^13^ All formol fixed paraffin embedded samples derived from patients who provided an informed consent form. The diagnosis was confirmed based on the 2017 World Health Organization (WHO) histological classification of OTs.^2^ A total of 15 CAm and 16 dental follicles (used as controls) samples were obtained (Supplementary Table 1).

### RNA extraction and cDNA microarray

Tissues (50 μm) were obtained from each sample.^14^ RNA extraction was performed using the ReliaPrep™ FFPE Total RNA Miniprep System (Z1002, Promega, Madison, WI, USA) according to the manufacturer’s instructions. Brief deparaffinization was performed using mineral oil at 80°C for 1 min, followed by the addition of 100 μL of lysis buffer, centrifugation at 10,000 ×*g* for 15 s, addition of 10 μL of proteinase K, incubation at 56°C for 15 min, and incubation at 80°C for 1 h. Then, 30 μL of DNase mix was added directly to the lower phase and incubated for 30 min at room temperature. In total, 325 μL of BL buffer and 200 μL of isopropanol (100%) were added to the lysed sample before vortexing and centrifugation at 10,000 ×*g* for 15 s. The entire lower (aqueous) phase was transferred to a binding column placed in a tube and the assembly was centrifuged at 10,000 ×*g* for 30 s, washed twice with 500 μL of 1× wash solution, and centrifuged at 10,000 ×*g* for 30 s. The centrifuge column was dried at 16,000 ×*g* for 3 min and the RNA was eluted in 50 μL of nuclease-free water centrifuge by centrifuging at 16,000 ×*g* for 1 min. RNA concentration and purity were determined using a NanoDrop ND-2000 spectrophotometer (Thermo Fisher, Rochester, NY, USA) considering only samples with >1.8 260/280 ratio. RNA integrity was evaluated using agarose gel electrophoresis. All ameloblastoma and dental follicle RNA samples were pooled into a single sample to obtain 2 μg RNA each for the synthesis of cDNA that was used for the microarray.

The cDNA from dental follicle control was labeled with Alexa 555, and that from CAm was labeled with Alexa 647, followed by mixing and hybridization with the GeneChip Human Mapping 10K Array (CHIP H10KA_07_38, AFFYMETRIX, Santa Clara, CA). The microarray data quantification of the chip images was analyzed using genArise software. A Z-Score cutoff of >2.0 was used to determine the upregulated genes. Then, R analysis was performed considering a Benjamini & Hochberg analysis with a false discovery rate (FDR) *p*<0.05 as significant. The microarray service of the Microarray Unit of the Cellular Physiology Institute of UNAM was used.^15^

### Functional enrichment analyses

The target gene list was submitted to DAVID 6.8, available online: https://david.ncifcrf.gov.^16^ Differentially expressed genes (DEGs), Kyoto Encyclopedia of Genes and Genomes (KEGG) pathway, and Gene Ontology (GO) enrichment analyses were conducted to analyze the functions of the candidate, mainly including biological process (BP), molecular function (MF), and cellular component (CC). Only elements of statistical significative with a *p*<0.05 were selected.

### Protein-protein interaction (PPI)

The Search Tool for the Retrieval of Interacting Genes (STRING); version 11.0, http://string-db.org/ database was used to predict the protein-protein interaction (PPI) networks of the DEGs.^17^ Then, the Cytoscape software was used to analyze the interaction with a combined score of >0.4 (http://cytoscape.org). Finally, the plugin molecular complex detection (MCODE) was used to screen the most significant module in the PPI networks with the MCODE score >, degree cutoff=2, node score cutoff=0.2, *k*-core 2, and max depth=100.

### Selection and analyses of hub genes

For the selection of the hub genes, those clustered with MCODE score ≥ 2.5 were selected, and then the effect of the hub genes on overall survival and disease-free survival was analyzed using the Kaplan-Meier plotter (KM plotter, http://kmplot.com/analysis) by adjusting the follow-up threshold to 60 months.^18^ To validate these hub genes, we used the Gene Expression Profiling Interactive Analysis (GEPIA Online: http://gepia.cancer-pku.cn/index.html) website to analyze data pertaining to RNA expression from thousands of samples from the Genotype-Tissue Expression (GTEx) and The Cancer Genome Atlas (TCGA) projects. To mimic the behavior of ameloblastoma to the maximum extent of the candidate genes, both analyses were adjusted for head-neck squamous carcinoma.^19^

### Real-time quantitative Reverse Transcription PCR (RT-qPCR)

Eight additional conventional ameloblastoma FFPE samples and three dental follicles as control were obtained from the Oral Medicine and Pathology Department and the Histopathological archive of the Oral and Maxillofacial Pathology specialty, ENES Leon, UNAM, their histological pattern was determined by two oral pathologists (Supplementary Table 2). The total RNA was obtained by using the ReliaPre FFPE Total RNA Miniprep System. Quantitative Reverse Transcription-PCR was performed using GoTaq 1-Step RT-qPCR System (Promega, Madison, Wisconsin, USA) according to the manufacturer’s instructions, and the data collection was performed on the ABI PRIS 7000 Sequence Detection Systems (Waltham, Massachusetts, USA). The primers for PDGFA were 5’-TTCTGGCTTTGTGTTTCTCCCTTA -3’ (sense) and 5’-TACGATTGGTTGACGCATAGTTCT-3’ (antisense); and for IL2RA were 5’- CAGGAACAGAAGGATGAATGAG-3’ (sense) and 5’- CCAATTAGTAACGCACAGGTAA-3’ (antisense); GAPDH primers were 5´-ACCACAGTCCATGCCATCAC-3´ (sense) and 5´-TCCACCACCCTGTTGCTGTA-3´ (antisense). Relative expression was computed using the 2^-(∆∆Ct) method.

### Immunohistochemistry assay and interpretation

The same eight additional conventional ameloblastoma FFPE samples and three dental follicles were employed for immunohistochemical analysis. Three poly-L-lysine-treated slides with 4-µm sections were obtained from each sample. The slides were deparaﬃnated and rehydrated conventionally in xylene and alcohol washes. Antigenic retrieval was performed in 10 mM citrate buﬀer in microwave heat (700 W for 3 min and 30 s). Endogenous peroxidase was inhibited with hydrogen peroxide at 3% for 20 min (Sigma-Aldrich, St. Louis, MO, USA). The background was blocked with 100 μl of 2% albumin for 20 min (Sigma-Aldrich, St. Louis, MO, USA), and 100 μl of 0.2% X-100 Triton was then added for 20 min (Sigma-Aldrich, St. Louis, MO, USA). The slides were incubated with primary antibodies for PDGFA (Santa Cruz Biotechnology, sc-9974, Santa Cruz, California, USA) and IL2RA (Santa Cruz Biotechnology, sc-665) at adjusted concentrations of 1:200 overnight at 4°C. Negatives controls were produced by the omission of primary antibodies and substituted with PBS as previously reported.^14^

After incubation, primary antibodies were removed and Immunodetector Biotinylated Link was subsequently added and incubated for 10 min (Bio SB, BSB 0007, Santa Barbara, California, USA). The slides were then incubated with Immunodetector HRP label for 10 min. Immunocomplexes were visualized via diaminobenzidine (DAB) incubation for 1 min, and the slides were counterstained for 1 min with Mayer’s hematoxylin. The slides were observed using a Leica DM750 microscope.

For the analysis of each marker, photomicrographs of 5 fields were obtained at 400× magnification from each sample using a Leica ICC50 HD camera. The intensity of staining (optical density) was obtained using the ImageJ software (NIH, Bethesda MD, USA); calibrating the quantification to establish the scale of optical density at: 0-0.9/negative, 1-1.9/mild, 2-2.9/moderate, and >3/intense.

### Statistical analysis

Statistical software of SPSS 26.0 (SPSS Inc., Chicago, IL, USA) was employed for the statistical analysis of data. The clinical-demographic data as age, gender, anatomic zone, and histological pattern were analyzed for descriptive distribution and central tendency (mean±standard deviation; x±sd). For the 2-ΔΔCt method, first we estimate the average Ct values for any technical replicates, then we estimate the delta Ct for each sample by the formula:


ΔCt=Ct (gene of interest) −Ct (housekeeping gene)

As a calibrator, we estimate average Ct. To calculate the ∆∆Ct values we employed the following formula:


ΔΔCt=ΔCt (Sample) −ΔCt (calibrator average)

The next step was doing the 2^-(∆∆Ct) of each sample to obtain the average 2^-(∆∆Ct) of control and ameloblastoma. The Shapiro-Wilk test was performed for immunohistochemistry results to determinate the normal distribution of data obtaining a *p*>0.05 not rejecting the null hypostasis, then an independent sample t-test was employed for the comparison between groups, considering *p*<0.05 as statistically significant.

## Results

The gender distribution was nine males and six females. The mean age was 37.8±17.7 years old. In total, 12 conventional ameloblastoma presented follicular and three plexiform patterns. All specimens were located in the mandible (Supplementary Table 1).

Although the quality of the RNA samples was high, the amount of RNA was insufficient to perform independent microarray analyses. Thus, the RNA samples from ameloblastoma and dental follicles were pooled to obtain ameloblastoma and dental follicle control groups, respectively.

### Identification of DEG gene ontology and KEGG pathway analysis

In total, 376 upregulated genes were identified. All genes were analyzed by the DAVID enrichment software, and the results of GO analysis indicated that 1) biological processes (BP) were particularly enriched in positive regulation of cell division, regulation of phosphatidylinositol 3-kinase signaling, branching involved in salivary gland morphogenesis, positive regulation of MAP kinase activity, positive regulation of mesenchymal cell proliferation, negative regulation of transcription from RNA polymerase II promoter, regulation of branching involved in salivary gland morphogenesis by mesenchymal-epithelial signaling, response to wounding, angiogenesis, fibroblast growth factor receptor signaling pathway, positive regulation of peptidyl-tyrosine phosphorylation, immune response, epidermis development, lung-associated mesenchyme development, Notch signaling pathway, response to drugs, induction of positive chemotaxis, epithelial tube branching involved in lung morphogenesis, negative regulation of apoptotic signaling pathway, positive regulation of GTPase activity, peptidyl-tyrosine phosphorylation, and protein localization to cell surface; 2) molecular functions (MF) were enriched in phosphatidylinositol-4,5-bisphosphate 3-kinase activity, Ras guanyl-nucleotide exchange factor activity, growth factor activity, protein tyrosine kinase activity, protein heterodimerization activity, heparin binding, protein phosphatase binding, protein domain specific binding, transcription factor binding, alpha-actinin binding, protein binding, platelet-derived growth factor receptor binding, enzyme binding, protein dimerization activity, and TATA-binding protein (TBP)-class protein binding; 3) cell components (CC) were enriched in the extracellular region, membrane, extracellular space, transcription factor complex, and flotillin complex (Table 1).

**Table 1:** GO analysis of differential expressed genes associated with ameloblastoma

Ontology	Term	Count	P-Value	FDR
BP	GO:0051781~positive regulation of cell division	6	0.00000412	0.0022802
BP	GO:0014066~regulation of phosphatidylinositol 3-kinase signaling	22	0.0000313	0.00865839
BP	GO:0060445~branching involved in salivary gland morphogenesis	4	0.000285	0.03155322
BP	GO:0043406~positive regulation of MAP kinase activity	15	0.000344	0.03171901
BP	GO:0002053~positive regulation of mesenchymal cell proliferation	4	0.00164912	0.13028075
BP	GO:0000122~negative regulation of transcription from RNA polymerase II promoter	8	0.00592798	0.36424165
BP	GO:0009611~response to wounding	4	0.00938832	0.45943785
BP	GO:0001525~angiogenesis	5	0.0148601	0.5106253
BP	GO:0008543~fibroblast growth factor receptor signaling pathway	4	0.01552901	0.5106253
BP	GO:0050731~positive regulation of peptidyl-tyrosine phosphorylation	4	0.01552901	0.5106253
BP	GO:0006955~immune response	6	0.0160365	0.5106253
BP	GO:0008544~epidermis development	4	0.01662072	0.5106253
BP	GO:0007219~Notch signaling pathway	4	0.02921914	0.80790927
BP	GO:0042493~response to drug	5	0.03324101	0.8244835
BP	GO:0043547~positive regulation of GTPase activity	6	0.04120035	0.9113518
BP	GO:0018108~peptidyl-tyrosine phosphorylation	4	0.04906818	0.96887935
CC	GO:0005576~extracellular region	17	0.000648	0.04653223
CC	GO:0016020~membrane	19	0.000745	0.04653223
CC	GO:0005615~extracellular space	15	0.00258885	0.10786879
CC	GO:0005667~transcription factor complex	9	0.00927696	0.28990486
CC	GO:0016600~flotillin complex	7	0.02007113	0.50177824
MF	GO:0046934~phosphatidylinositol-4.5-bisphosphate 3-kinase activity	7	0.0000111	0.00162983
MF	GO:0005088~Ras guanyl-nucleotide exchange factor activity	7	0.000126	0.00927298
MF	GO:0008083~growth factor activity	7	0.000468	0.02292204
MF	GO:0004713~protein tyrosine kinase activity	6	0.00329741	0.1082232
MF	GO:0046982~protein heterodimerization activity	8	0.00368106	0.1082232
MF	GO:0008201~heparin binding	6	0.00552787	0.13543269
MF	GO:0019903~protein phosphatase binding	3	0.00883842	0.18560683
MF	GO:0019904~protein domain specific binding	6	0.01132918	0.2081737
MF	GO:0008134~transcription factor binding	6	0.02576728	0.40442461
MF	GO:0051393~alpha-actinin binding	4	0.0288818	0.40442461
MF	GO:0005515~protein binding	29	0.03026307	0.40442461
MF	GO:0005161~platelet-derived growth factor receptor binding	4	0.03325244	0.40734242
MF	GO:0019899~enzyme binding	6	0.03860665	0.43655217
MF	GO:0046983~protein dimerization activity	5	0.0447642	0.45324775
MF	GO:0017025~TBP-class protein binding	4	0.04624977	0.45324775

KEGG analysis data (Table 2) showed that the upregulated genes were enriched in pathways in cancer, PI3K-Akt signaling, and Jak-STAT signaling pathways.

**Table 2:** KEGG pathway analysis of differential expressed genes associated with ameloblastoma

Term	Count	P-Value	Genes	FDR
hsa05215:Prostate cancer	7	0.00337144	PDGFA, CTNNB1, ERBB2, FGFR1, FOS, DVL2, FZD4	0.15462207
hsa04015:Rap1 signaling pathway	6	0.00554887	PDGFA, CTNNB1, VEGFD, FGF7, FGFR1, PAK1	0.15462207
hsa04151:PI3K-Akt signaling pathway	9	0.005947	IL2RA, GH1, PDGFA, VEGFD, FGF7, FGFR1, NDP, IKBKG, AKT3	0.15462207
hsa05200:Pathways in cancer	12	0.01022767	PDGFA, CTNNB1, VEGFD, FGF7, FGFR1, ERBB2, POU5F1, GREM1, NBL, FZD4, GRB2	0.19943962
hsa04630:Jak-STAT signaling pathway	9	0.01342465	IL2RA, GH1, IL13, IL11RA, MPL, THPO, THBD, STAT1, JAK3	0.2094245
hsa05218:Melanoma	4	0.02504124	PDGFA, FGF7, FGFR1, PIN1	0.27903096
hsa04520:Adherens junction	3	0.02504124	CTNNB1, ERBB2, CD70	0.27903096
hsa04510:Focal adhesion	8	0.03371729	PDGFA, CTNNB1, FGFR1, VEGFD, ERBB2, THPO, THBD, MRC2	0.3287436
hsa04014:Ras signaling pathway	5	0.04260335	PDGFA, VEGFD, FGF7, FGFR1, TIAM	0.36922906

### PPI network and modular analysis

**Figure 1:**
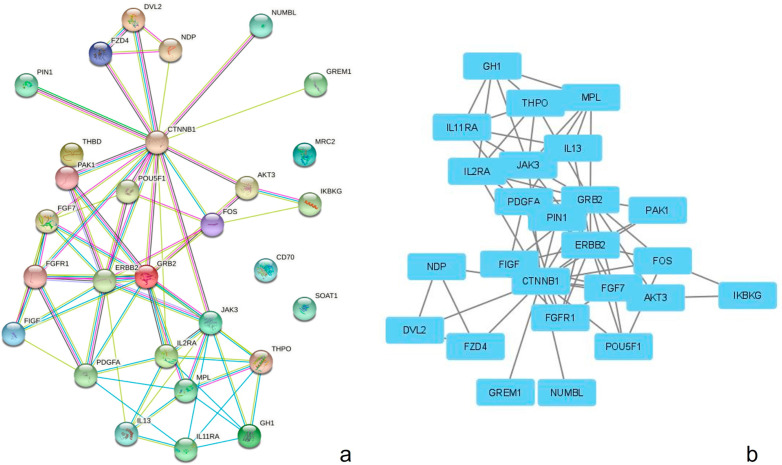
PPI of the differentially expressed genes constructed using STRING online database and Cytoscape software analysis. a) A total of 31 differentially expressed genes were identified in the network. b) Module analysis via Cytoscape software (degree cutoff=2, node score cutoff=0.2, k-core=2, and max depth=100)

A total of 31 DEGs, including 29 nodes and 68 edges, were imported into the PPI network complex. We then applied Cytoscape MCODE for further analysis, and the results showed 25 nodes and 68 edges, with 20 clustered genes with an MCODE score >2.5 (Figure 1).

### Analysis of core genes using Kaplan-Meier plotter and GEPIA

**Figure 2:**
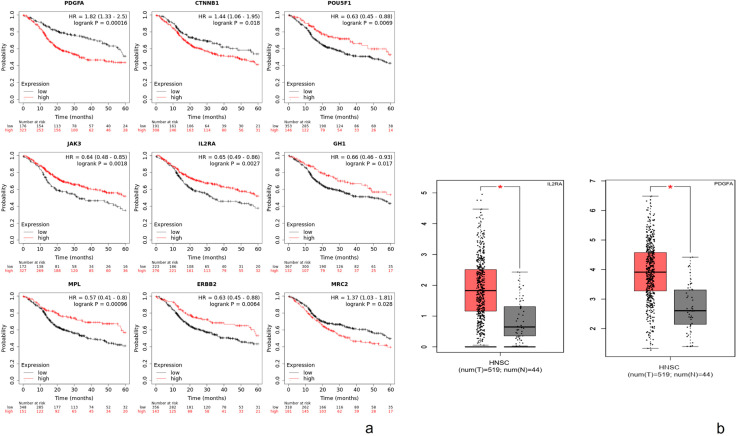
A) Prognostic information of the 20 core genes. Kaplan-Meier plotter online tool was used to analyze the prognostic information and nine genes were found to be significantly associated with survival rate (*p<0.05). B) Validation of the significant genes by GEPIA. The significant genes expressed in patients with ameloblastoma were compared to those in healthy individuals. Only platelet-derived growth factor A (PDGFA) and interleukin 2 receptor subunit alpha (IL2RA) showed significant differential expression (*p<0.05)

**Figure 3:**
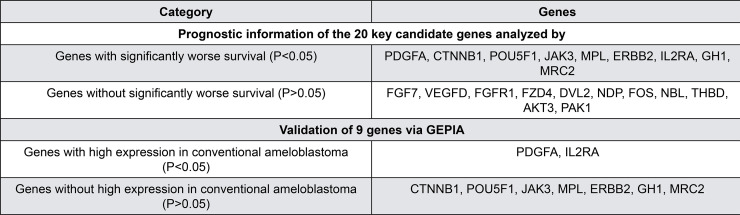
Selection of candidate genes

The Kaplan-Meier plotter was used to identify the survival data for these 20 clustered genes. Only nine genes were significantly associated with poor survival (Figure 2). GEPIA was used to validate these nine genes and led to the identification of two genes (platelet-derived growth factor A (PDGFA) and interleukin 2 receptor subunit alpha (IL2RA) with significant correlation (Figure 3).

### IL2RA and PDGFA gene expression and immunohistochemistry analysis

**Figure 4:**
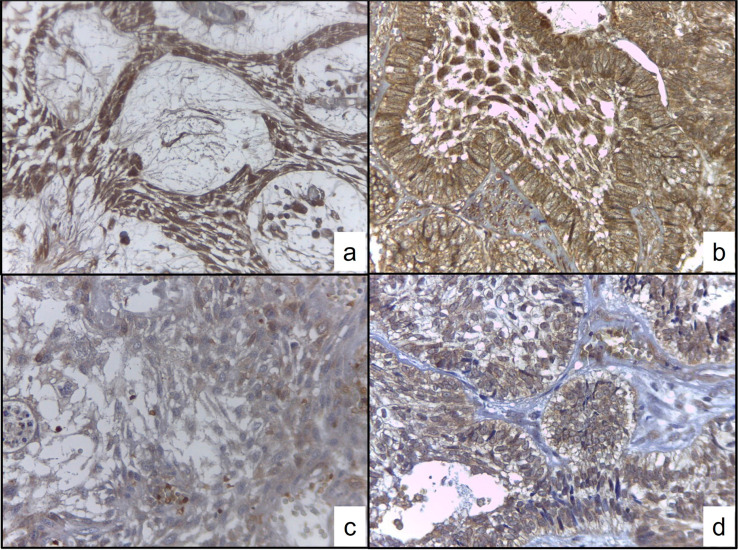
Immunoexpression analysis. a) and b) showed IL2RA intense immunoexpression in stroma and parenchyma of conventional ameloblastoma. c) and d) showed more selective immunoexpression of PDGFA for parenchyma

Three samples showed a follicular pattern and five were plexiform (Supplementary Table 2). The gene expressions of IL2RA and PDGFA in conventional ameloblastoma were higher than those in the dental follicle in the 2^-(∆∆Ct) method relative quantification with 362±66 and 419±33 measure units, respectively. The immunoexpression analysis showed that IL2RA presented an intense expression in the parenchyma and stroma of CAm, especially in the follicular pattern. The PDGFA showed a moderate to mild immunoexpression in plexiform and follicular patterns respectively, predominantly in the parenchyma, however, there was no significant difference related to histological pattern (Figure 4).

## Discussion

Conventional ameloblastoma is a benign epithelial odontogenic tumor that is frequently diagnosed in young adults with a median age of 35 years without any gender-specific trend. CAm often progresses slowly but is locally invasive. Untreated tumors resorb the cortical plate bone and extend into the adjacent tissue.^5,20^ Our samples used for microarray were obtained from 15 patients (six females and nine males) with a mean age of 37.2±17.8 years, which is consistent with that reported previously.^20^ However, it has been proposed that when mutation BRAF V600E is present, the presentation age is earlier than the wildtype genotype.^2^ In Mexico, CAm is commonly diagnosed in advanced stages due to the absence of symptoms and low prevalence, which results in detrimental effects on the bone as described above, thus complicating the patient’s treatment and prognosis.^3^ Increasing our knowledge of the mechanisms underlying the pathogenesis of ameloblastoma is necessary to improve the prognosis and treatment approaches of patients with CAm, as well as the number of possible therapeutic targets. Signaling pathways of WNT, Akt, and FGFR1, effects on bone remodeling by RANK-RANKL and OPG, degradation of extracellular matrix by MMP, and mutations in BRAF and SMO are all molecular events associated with CAm pathogenesis, however, the high-throughput assay could improve this knowledge.^2,7,8^

The application of bioinformatics methods on microarray profile datasets is an important strategy to identify more useful therapeutic and/or prognostic biomarkers of ameloblastoma. A total of 376 upregulated genes (log FC>2) were identified. We performed GO function and KEGG pathway enrichment analysis using the DAVID platform to understand the functional relevance of these DEGs and found that, for biological processes, molecular functions and cell components of the upregulated genes were particularly enriched in positive regulation of cell division, positive regulation of MAP kinase activity, positive regulation of mesenchymal cell proliferation, growth factor activity, protein tyrosine kinase activity, and other elements related to cancer development. These data reinforce that although ameloblastoma is classified as a benign tumor, molecularly it emulates the patterns observed in malignant neoplasms, such as gastric cancer, nasopharyngeal carcinoma, breast, or prostate cancer.^21-24^Hu, et al.^10^ (2016) in their pathway and gene enrichment analysis showed that inflammation, MAP kinase, and cell cycle regulation are differentially expressed, however when separate the expression of pre-secretory ameloblast and odontoblast contrasting differences was observed. In high-throughput analysis as microarrays, the separate analysis of tumoral parenchyma and stroma via laser capture microdissection could bring information that is validated when immunohistochemistry is performed for protein determination and Nanostring gene expression analysis.^10,11^ In our approach, the RT-qPCR and immunohistochemistry were performed, despite not having separated the parenchyma from the stroma since the microarray analysis. Our methodology provides us the possibility of observing and analyzing the tumor microenvironment in a comprehensive way, in which the most relevant elements stand out, and after their validation with a more sensitive assay, as above mentioned, the results could be correlated directly with clinical variables.

We built a PPI network complex with 29 nodes and 68 edges using the STRING online database and Cytoscape software. Twenty upregulated hub genes were screened from the PPI network complex by Cytotype MCODE analysis. Moreover, the Kaplan-Meier plotter analysis revealed that nine of the 20 genes were significantly associated with poor survival. Validation of these nine genes led to the identification of two genes that showed high and significative expression (P<0.05) compared to normal samples by GEPIA analysis (PDGFA and IL2RA) and these represent potentially new effective targets to improve the prognosis or treatment of CAm. Our bioinformatic protocol or algorithm is based on the genome-proteome-clinical utility premise; using GO and KEGG to assess the enriched genome panorama; with protein-protein interaction to estimate the proteome status; and with Kaplan Meier and GEPiA analysis to search the relationship with survival. The hub genes were validated by RT-qPCR and immunohistochemistry to confirm the high presence and observe the distribution in tumoral parenchyma and stroma. Although these techniques only allow the detection of a single candidate per test, they offer greater sensitivity; and the possibility to establish their association with cytological or histological criteria.

PDGF promotes cell proliferation, survival, and migration. Alterations in signaling have been observed in cancer, fibrosis, and atherosclerosis. PDGF is an important factor in ameloblastoma pathogenesis and the expression of the PDGF chain is higher in ameloblastic tumors than in tooth germs, and alongside its cognate receptor (PDGFRA) it is expressed at a variable level in ameloblastomas.^25,26^

PDGF signaling is important for the growth and differentiation of stem cells, particularly mesenchymal cells. Their dimerization promotes autophosphorylation at ten sites, which can interact with SH2-domain-containing signaling proteins. Activated signaling proteins include phospholipase C, PI3K, Grb2, and others. Binding these proteins leads to the activation of several signaling pathways as MAP kinase pathways, PI3-kinase-Akt, and PLC pathways.^23^If we consider PDGFA as a possible therapeutic target, there are three main approaches to inhibit the PDGF/PDGFR pathway: 1) sequestering the ligand with neutralizing antibodies, 2) blocking the receptor with receptor-specific antibodies or small molecules inhibitors, and 3) blocking the kinase activity of PDGFR using low molecular weight inhibitors. Olaratumab, nilotinib, dasatinib, ponatinib, sunitinib, imatinib, and other anti-PDGF drugs have shown significant clinical results in many malignancies. A similar strategy has been developed for BRAF V600E mutation-positive ameloblastomas, in which treatment with vemurafenib showed significant clinical results.^27^ Many reports postulate that the aggressiveness and frequency of positive BRAF V600E ameloblastoma are high.^28-31^

Cytokines are major mediators of the immune response. Cytokine ligands and receptors control various cellular functions, including proliferation, differentiation, and cell survival/apoptosis of leukocytes; however, they are also involved in many pathophysiological processes. The interleukin-2 receptor is involved in the regulation of immune tolerance by controlling regulatory T cell (TREG) activity. The interleukin 2 (IL2) receptor alpha (IL2RA), beta (IL2RB), and gamma chain (IL2RG) constitute high-affinity IL2 receptors. Homodimeric alpha chains (IL2RA) result in a low-affinity receptor, whereas homodimeric beta (IL2RB) chains produce a medium-affinity receptor.^32^ Ameloblastoma cells and surrounding stromal cells, such as fibroblasts, may contribute to ameloblastoma pathogenesis.^8^Chantravekin and Koontongkaew conducted a co-culture test and three-dimensional organotypic culture and showed the role of the stroma on the tumor parenchyma, as fibroblasts associated with ameloblastomas modulated tumor development, promoting the proliferation and invasion of tumor cells.^33^ It has been hypothesized that ameloblastoma cells and stromal fibroblasts may be reciprocally activated via cytokines, such as IL-6, IL-8, IL-1a, and recently IL33, to create a tumoral microenvironment that promotes tumor formation.^8,34^ Damoiseaux^35^ (2020) has reported that the IL2RA fraction can function in a diverse way to lead to leukocyte activation via paracellular or even soluble forms, which can affect the functionality of cells such as cytotoxic CD4 and CD8 T lymphocytes at the tumor level. This is the first report to identify IL2RA as a possible participant in the mechanism underlying the development of ameloblastoma. When we analyzed the KM plot result, we observed that patients with a high level of IL2RA have a greater probability of survival. If we correlate this result with immunoexpression results, immunomodulation by IL2RA is present, but additional studies would need to verify the mechanism that conducts it, as well as the result of PDGFA, since due to its greater tendency to express itself in tumor parenchyma, it becomes a direct target. Nevertheless, the validation analysis of IL2RA and PDGFA by immunohistochemistry reinforces the concept that the parenchyma and stroma relationship is a necessary feature that must be considered to improve our understanding, in order to develop better therapeutic strategies. Zhang, et al.^36^ (2022) suggested in a bioinformatic analysis that macrophages could infiltrate the ameloblastoma and participate in their pathogenesis.^36^ That could be a relationship mainly with IL2RA, however, to prove this immunological relationship their validation is necessary.

Taken together, our bioinformatic analysis identified two hub genes (PDGFA and IL2RA) between CAm and normal dental follicles. The results suggested that these genes play key roles in the pathogenesis, progression, and prognosis of CAm. The main limitation to postulating PDGFA and IL2RA as therapeutic targets is the verification of their reach in cellular or animal models, in which the biological behavior could be measured and correlated. For this reason, identifying how we can affect CAm in these specific targets to provide useful information on these new biomarkers is necessary.
